# Ascribing Sentience: Evidential and Ethical Considerations in Policymaking

**DOI:** 10.3390/ani12151893

**Published:** 2022-07-25

**Authors:** James William Yeates

**Affiliations:** World Federation of Animals, Boston, MA 02130, USA; james.yeates@wfa.org

**Keywords:** animal welfare, cephalopod, decapod, Nolan principles, policymaking, sentience, suffering

## Abstract

**Simple Summary:**

Policies that affect animals need to consider which animals can experience suffering and other positive or negative feelings. Policymakers, therefore, need to determine which animals should be treated as sentient. This requires answering several questions about the definition, criteria, evidence requirements and sources, and how far the conclusions should be generalized. These should use scientific evidence where available, but the process is also full of ethical aspects that should conform to ethical principles applicable to policymakers, such as selflessness, objectivity, accountability and openness.

**Abstract:**

Deciding which animals are sentient is an important precursor for decisions about the application of animal welfare legislation, and the wider assessment of the impacts of policies on animal suffering. We ascribe sentience in order to inform decisions about how animals should be treated, and how their treatment should be regulated. This ascription is both an ethical and an evidential process, and what evidence to use and require are ethical questions. Policymakers, therefore, cannot simply rely on scientific evidence in an ethically neutral way, but must be conscious of the ethical assumptions and positions underlying the process of ascription and its application in policy and law. As such, ethical principles that apply to policymaking apply to the ascription of sentience. This paper considers the implications of the Nolan principles for public service on the ascription of animals.

## 1. Introduction

Sentience–the ability to experience positive and/or negative feelings–is an important question in contemporary policymaking. The ascription of sentience to animals is a matter of public interest (e.g., the recent television show *My Octopus Teacher*), and academic enquiry e.g., [1,2,3], with a dedicated journal, *Sentience*. It is also an ethically relevant question insofar as the ascription of sentience effectively constitutes an “entry ticket” to being morally considered. Similarly, government and intergovernmental policies in relation to the ascription of sentience to animals are important because they affect how the treatment of animals is regulated by further policies and legislation such as the European Union Treaty of Lisbon in 2007.

The attribution–or denial–of sentience is of vital importance for animal-related policies. For animals, it affects whether their welfare has legal protection or even whether it is considered within policymaking at all, which in turn affects how they are treated and the degree to which they suffer. For humans, it affects the determinants and restrictions of behaviour that have the potential to be immoral, unsustainable or, conversely, profitable or enjoyable. If policies attribute sentience incorrectly, they could either allow or cause suffering of sentient animals or unnecessarily limit the legitimate use of insentient resources. Which animals are deemed to be sentient within policy frameworks is therefore an important component of the impacts of animal welfare and other policies.

As an example, the UK has recently engaged in political debates e.g., [4] and policymaking (e.g., UK Animal Welfare Sentience Act 2022) in relation to recognising animal sentience, in connection with its exit from the EU. This latter instrument also recognised the sentience of cephalopod molluscs and decapod crustacea, in line with recent research and reviews that concluded that they should be deemed, and treated as, sentient e.g., [5,6]. As such, policymakers in this process included a variety of agents: politicians, civil servants, scientists and experts, with a variety of roles in reviewing evidence, making recommendations, and drafting and passing legislation.

The UK’s recent debates have been conducted in the context of the existence of agreed ethical principles for policymaking, the UK government’s Seven Principles of Public Life (also known as the Nolan Principles): selflessness, integrity, objectivity, accountability, openness, honesty, and leadership. These standards were first set out in 1995 in the first report on the Committee on Standards in Public Life [7] and were amended in the Committee’s 14th and 23rd reports [8,9]. They are now included in a range of codes of conduct across public life and are applicable to policymakers and others who work as a public office-holder (logically including those appointed as scientists on such issues) [10]. They outline expected ethical standards and are generally accepted as basic norms [11]. Although some have questioned their adaptability [12] and postulated that we are in “a post-Nolan age” [13,14], others have reaffirmed the value of their recognition [15].

This paper considers some of the questions for policymakers–conceived broadly–to answer when deciding which animals to protect as sentient, highlighting the importance of elucidating and determining the underlying ethical and conceptual concerns. This paper is primarily an argument for attention to be given to the fact that many ethical considerations are implicitly included in the ascription of sentience.

In light of this, this paper is further an argument for policymakers to ensure that definitions and norms for the ascriptions of sentience are explicit and both evidentially and ethically justifiable. As ascriptions form part of the policymaking process in relation to animals, the general principles such as Nolan principles that apply to policymaking are applicable (and should be applied) to the methods of ascribing sentience to nonhuman animals. So, this paper then illustratively considers the implications of the Nolan principles on the ascription of animals, referencing the recent consideration of the sentience of cephalopods as examples. While the Nolan principles and recent debates are UK-based, the considerations are applicable to relevant policymaking more widely.

## 2. Essential Questions for Policymakers in Ascribing Sentience

While the question of which animals are sentient may seem simple, it is important to understand exactly what is being asked in the policymaking process. In fact, when the question is operationalised, it can be seen as a combination of several questions that it is useful to separate. These might be categorised as questions of definition and criteria; questions of source; questions of evidence; and questions of generalisation.

### 2.1. Questions of Definition and Criteria–Deciding What Evidence “Counts”

A good place to start is to ask what sentience is and can be operationally assessed.

An obvious question is what definition to use. In the first place, what are we referring to when we ask if an animal is sentient? As well as the basic question of what sentience ‘is’, we have secondary questions about how narrow or broad (and how vague or precise) a useful definition should be (e.g., in relation to the ability to have feelings or only certain feelings). If a definition is too narrow and precise then policies will exclude animals from needed protection a priori. If a definition is too broad, policies could unnecessarily prevent the protection of sentient animals, for example if they were to protect insentient pathogens at the expense of sentient patients. If the definition is too vague, then policies risk contention and confusion about the very task of ascription.

A second obvious question is which observable criterion or criteria are relevant to an ascription of sentience. While ascriptions might be based on metaphysical or ontological assumptions (e.g., a presupposed scale naturae based on Thomistic theology), secular policymaking generally requires a systematic and scientific process for ascribing sentience. The attribution of sentience also faces background questions of whether animals’ feelings, as subjective states, are ones that we can directly observe or quantify. However, it appears to be generally assumed within the policies under discussion that we can at least infer animals’ feelings (e.g., “suffering” within the Animal Welfare Act 2006).

We need justifiable ways to assess which data we should count and which we should discount. We face the most salient question of what criteria constitute evidence of sentience (or of insentience, although arguably there is no such thing as evidence of insentience, only a lack of evidence of sentience or evidence that throws doubt on evidence for sentience). Behind this question are more technical questions such as how theoretically-laden and specific to be in developing our criteria [16].

Methods of ascription might draw analogies that compare animals to humans, assuming associations between observable features and unobservable experiences. This raises questions of which properties need to be similar, and how similar they need to be, for an animal to be deemed sentient. To answer this, we might turn to abductive reasoning that identifies best explanations of behaviour that involve experiences, but scientists and philosophers have proven highly versatile in generating explanations (of varying plausibility and practical applicability) that do not require experiences as part of the explanation.

As a specific example, policymakers might base ascriptions partly on analogies between human and other animals’ central nervous systems. However, this leaves how we define (sufficiently) humanlike open. There is evidence that central neocortices are associated with sentience in ourselves, but this does not mean they are a necessary or a sufficient criterion. It is certainly conceivable that the very different neural structures of cephalopods might be sufficiently complex to support feelings despite their dissimilarities to humans [17]. As another example, we might consider that how closely animals are evolutionarily related to humans (their phylogenetic proximity) supports the belief that they are sentient, on the assumption that sentience is conserved through evolution. However, this says nothing about whether sentience might be evolutionarily ancient (and therefore shared across a wide taxon), or the result of convergent evolution (and therefore shared by multiple taxa).

Policymakers might use many candidate criteria. The DEFRA assessment of cephalopod and decapod sentience used a combination of neurological and behavioural criteria adapted and improved from [18], see also [19]. The SAWC assessment used a combination of neurobiological, behavioural and phylogenetic criteria. However, if we use multiple criteria, this raises a third question.

The third question is how important different criteria are. While no criterion is necessary or sufficient for proof, they may be more or less convincing. For example, the DEFRA-commissioned study proposed that the criterion that animals value a putative analgesic or anaesthetic when injured in particular ways “provides particularly compelling evidence in its own right” whereas the criterion of possessing nociceptive receptors sensitive to noxious stimuli “could only ever form a small part of a wider case for sentience, due to the difference between sentience and nociception” (although it was unclear how their schema differentiated between criteria as not having equal significance).

Often, we assess the criteria with reference to how specific and sensitive they appear to be. The DEFRA-commissioned assessment notes that “Our criteria are not unreasonably demanding (they are not demands for absolute certainty). This can be seen by noting that well-researched mammals, such as lab rats (*Rattus norvegicus*), would satisfy all of them [20]. At the same time, the criteria are also rigorous and robust. This can be seen by noting that cnidarians (jellyfish and sea anemones) would not convincingly satisfy any of the criteria on the basis of current evidence [21,22,23,24] of which we are aware.” [6]. However, in the absence of a “gold standard” of proof, it is obviously insufficient to assess methods solely in terms of which animals they imply to be sentient, since this is exactly the issue in question. 

### 2.2. Questions of Source–What Evidence to Consider

Another key question for policymakers is what source(s) of evidence are relevant for assessing sentience. In everyday life, we use direct intuition or perceptions (e.g., for one’s own pets or family-members), and such views might be collated and assessed e.g., [25]. However, policymaking in many countries tends to require scientific evidence of feelings. For example in the UK, the Animal Health and Welfare (Scotland) Act 2006 (s16(4) and Animal Welfare Act 2006 (s 1(4)) allow Ministers to include animals under protection if (and only if) they are ‘satisfied, on the basis of scientific evidence, that creatures of the kind concerned are capable of experiencing pain or suffering’. Policymakers will talk of basing decisions on science and debates may reject more “anecdotal” or “subjective” assessments. Sentience is often seen as a natural property to be investigated within empirical paradigms, such as cognitive and animal welfare sciences [26], and policymakers often draw on scientific evidence [27]. Scientific approaches apply inductive methods that look for evidence and observations through standardised evidence-gathering methods.

However, the decision to use scientific evidence in making ascriptions is not inevitable: it is a decision. Indeed, it is one that is not ethically neutral insofar as it involves decisions to endorse the assumptions of a scientific approach (e.g., placing a threshold for evidence) and insofar as it implies an endorsement of the implications of that approach (e.g. the policies that apply if sufficient scientific evidence is lacking). The inherent ethical components underlying scientific questions has been well recognised for decisions about how animal welfare scientific data should be used to decide how sentient animals should be treated [28,29]. It is equally true for decisions about which animals are sentient.

Ascribing sentience is not a simple matter of science or fact. It is a policy question. It is about what animals we treat as sentient, and it is about how we decide to make ascriptions. As a policy question it has both an evidential and a normative basis. What evidence exists, and its reliability, are evidential questions that can be addressed scientifically. However, what evidence to use and acquire, and how and how far to apply it, are ethical questions. These questions not only have ethical implications in how we treat animals, but our method of ascription is itself an ethically-loaded process on which we should decide. The methods of attribution prescribed in policies are essentially normative. Policies’ definitions and ascriptions happen (and should happen) inside an ethical framing, which occurs when scientists or policymakers create frameworks for ascription of sentience (which may be more or less conscious to the investigators).

Deferring to scientific evidence requires recognition and acceptance of the consequences of that deferral as a de facto decision on how animals can be treated. Policymakers, therefore, cannot simply draw on scientific evidence in an ethically neutral way, but must be conscious of the ethical assumptions and positions underlying the process of ascription and its application in policy and law. The application of science cannot absolve policymakers of responsibility for the implications of a scientific approach.

### 2.3. Questions of Evidence–Deciding How Much Evidence Is “Enough”?

In practice, there is often varying amount of evidence to support an ascription of sentience. Science is a process, and the use of scientific data relies on the studies being conducted and researched. Evidence therefore provides support to varying degrees, without ever constituting a definitive proof against strict inclusion/exclusion criteria. This support is partly a matter of the number and power of studies, but complicated also by the frequent variety of types of evidence, for example if a species exhibits multiple different forms of associative learning in different contexts, where there might be different levels of evidence for each.

In particular, policymakers often lack evidence not because of any properties of the animals themselves, but because scientific studies have simply not been done, or have been done at an insufficient power to reject their null hypotheses. For example, there have been several studies on octopods and true crabs in comparison to other cephalopods (e.g., nautiloids, squid and cuttlefish) and decapods (e.g., penaeid shrimps and anomuran crabs) and the weight of evidence is in proportion to the scientific attention devoted to each taxon or species [6].

Policymakers need to answer three key questions in relation to how much evidence is needed:

The first question is whether the policies need to be framed as binary categories of (treated as) “sentient” vs. “insentient”. This approach is tempting insofar as it tries to mirror what we may assume is the ontological reality that animals either are or are not able to experience feelings. However, it does not reflect the discreteness and complexity of the evidence, where many criteria admit of degrees (e.g., similarity of behaviour, neurobiological function or phylogenetic proximity). We need a way to deal with such continuous data without oversimplification.

The second question for policymakers is specifying what weight and strength of evidence is sufficient–or satisfising–to ascribe sentience (or insentience) against the given criteria. For example, if it is considered that self-administration of analgesics is a criterion, how much evidence is needed to demonstrate self-administration: a single observation, a single study (in which case, at which confidence level), a series or a meta-analysis. This is even more complicated when considering the cumulative evidence across multiple criteria. Even when there is evidence, there is the potential for disagreement over whether the available evidence is sufficient, and an unavoidable risk of some people wanting to call for more (or different) evidence (hopefully in good faith).

Decisions about the threshold of what evidence is sufficient have effective and practical implications, particularly insofar that animals for which there is “insufficient” evidence can be treated in certain ways that would be considered unethical if they were deemed sentient. Under the UK’s Animal Welfare Act 2006 and Animal Health and Welfare (Scotland) Act 2006, whether ministers are satisfied that animals are of a kind capable of experiencing pain or suffering, affects whether they are then protected against unnecessary suffering and whether those responsible for them are required to adequately meet the needs of (sentient) animals in their care, whereas those that are not aere excluded from the protection afforded by that legislation.

The decision on what counts as “sufficient” cannot be a purely scientific question but one that relies on a metacognitive (and non-statistical) assessment of the evidence. Generally, science cannot *prove* facts per se, it can only reach degrees of confidence in an association, theory or belief. More specifically, it is arguably impossible for there to be enough evidence to prove sentience (except in oneself at a particular time). The same logic means we cannot prove insentience either.

This explains why there is the potential for disagreement even in the face of similar evidence. For example, some cephalopods have been considered sentient in the UK’s Animals (Scientific Procedures) Act (Amendment) Order 1993 and the Welfare of Animals (Transport) (England) Order 2006 but were not included as (sentient) animals under the Animal Welfare Act 2006 and Animal Health and Welfare Act 2006. As the latter were subsequent or contemporaneous with the former, these differences cannot have been due to the evidence available (first-hand evidence aside), but on the use of that evidence.

A third question is what the policy should be in the absence of sufficient evidence. Assuming we have a definition and evidential criteria for ascribing sentience, policymakers need to decide what default policy should be followed in the absence of enough data to the contrary. Do we assume insentience (or at least that animals should be treated as insentient) in the absence of sufficient evidence for sentience, or do we assume sentience (and the associated degree of protection) in the absence of evidence that warrants sufficient doubt? In essence, policymakers need to choose whether to place the “burden of proof” on ascribing sentience or on refuting it. Obviously, this decision has implications for all cases where there is insufficient evidence to meet the requisite burden. For example, Cephalopods were afforded “the benefit of the doubt” by the Animals Procedures Committee proposed in 1992 (Section 3), whereas decapods were assumed insentient until further evidence was obtained.

In the policymaking context, this default policy may not explicitly relate to an ascription. It might be framed entirely in terms of other policy areas, for example if animals deemed insentient are therefore outside the scope of animal welfare protection policies, this means that their protection is not an exception to wider policies of how animals are treated. In particular, it might be that humans are allowed to treat such animals in any way they like (i.e., in the absence of prohibitions, such behaviour is, ceteris paribus, permitted) or in ways that conserve the historic level of protection (which is often minimal, and therefore similarly permits ongoing harms). In the case of cephalopods and decapods, this would affect whether their use would come under the relevant regulations relating to animals in scientific procedures, farming, wildlife and welfare at the time of killing. If not, the research, farming, management or slaughter may be permitted using methods that are otherwise permissible (or even subsidized in some cases).

### 2.4. Questions of Generalisation–Deciding How Far to Apply Ascriptions

When there is relevant and sufficient evidence for some animals to be considered sentient, policymakers also need to decide how far to generalise across taxa and ages. Are ascriptions applicable only to fully mature adults, or to certain earlier developmental stages or chronological cut-off points? Are they applicable at the level of species, genera, classes, infraclasses, families, phyla or kingdoms? It seems hard to avoid arbitrary policies without valid rules of generalisation. The risks are parallels to the risks related to the choice of criteria and evidence: over-generalisation might include animals who are insentient (essentially the problem of “marginal cases”); under-generalisation might exclude sentient animals (as seen in human exceptionalism).

The first question is about how far we can legitimately generalise in the absence of data relating to all species or development types. Very few of the millions of species and billions of animals (including very few crustacea, cephalopods or even vertebrates-and, for that matter, few individual humans) have been scientifically studied against recognised criteria of sentience. The fundamental issue underlying this question is that observable features, such as neuroanatomy, physiology and biochemistry are partly but incompletely conserved across taxa (singles-species taxa aside, although members still have some phenotypic and ontogenic variation). The wider the generalization across more diverse animals, the lower the shared degrees of similarity and the weaker the argument for such widespread ascriptions. Before obtaining data, policymakers may decide how widely they are willing to extrapolate data across groups.

The question of generalisation may be taken, at least partly, as prior to the evidence-gathering. This is firstly seen in terms of the scoping of each analysis, for example the SAWC opinion considered only cephalopods [5]; the DEFRA-commissioned study considered only cephalopods and decapods [6]. It is secondly seen in decisions concerning whether analyses will accept data collectively from any members as representative of the whole taxon. Although very few crustacea and cephalopods have been studied against recognised criteria of sentience, Birch and colleagues [6] considered it appropriate to extrapolate to all decapods and cephalopods but not to other crustacea or mollusks.

The second question concerns how generically criteria are specified or interpreted in the face of interspecific differences. This question represents a philosophical conundrum if the model for ascribing sentience is based on generalisations from ourselves (as the one species some of which we can each confidently deem sentient). The *specifics* of observable characteristics in humans might seem “accidental” and applying them too narrowly in scope (especially if treated as necessary criteria) might make them too restrictive to usefully inform ascriptions of sentience. The wider the generalisation, the greater the likelihood of similar functions with different features or vice versa, which may be the result of convergent or divergent evolution.

More precisely, the extrapolation is from oneself, as the one individual we can each confidently deem sentience (which we might call “egomorphism” [30]). However, for most of us, many of the features that one might associate with sentience (e.g., neuroanatomy) are based on reverse comparisons with other humans (e.g. those who have had anatomical or physiological studies to which I, for one, have not been subjected but who show behavioural evidence of sentience and otherwise show similar morphology to oneself). I assume I have a cerebral cortex because other humans (and primates etc.) do. This essentially means the generalization challenge is two-way.

As one example, within the Animal Welfare Act 2006, policymakers specified the limits of generalisations as a single criterion–being a vertebrate–thereby excluding invertebrate cephalopods. However, this criterion is not particularly generic insofar as it relies on a specific neuroanatomical/phylogenetic criterion for ascribing sentience: the presence of a spinal column, i.e., a skeletal and neural linear structure (hagfish are often included in vertebrata or craniata, although they lack true backbones, but it is not clear if they are included under the scope of the legislation, and it is assumed that the legal decision would be based on the scientific debate as to their taxonomy or anatomy), when it is entirely conceivable for sentience to correlate to other neural structures. As another example, invertebrates may have different neurotransmitters and so their behaviour may be modulated by different pharmacological tests (e.g., opioid analgesics). The more specific the features described, the narrower the generalization can be expected to be, and thus the narrower the ascription of sentience and protection–and, conversely, the wider the “default” policies will apply to those animals beyond the generalization.

Scientific data are important for determining observable associations and how widely they can be generalised across taxa. This implies that taxa are not sufficient or necessary criteria for absolute general ascriptions, which would mean that all or only animals in a given clade are sentient. (If I suddenly learnt I was a species of crustacean, I would not want that to affect how others ascribe sentience to me, and nor, if I learnt my fellow colleagues were crustacean too, would I suddenly be more sceptical of their sentience). Taxa are practically useful in making predictive, inductive generalisations about how widely *observable* evidence can be expected to be found (e.g., the predictability of members of a taxon to have similar neurology or exhibit similar behaviour). This avoids assuming that a clade maps precisely onto an inductive rule of ascription, since it allows us to recognise where individual members of a clade do not show that evidence (e.g., “marginal cases” such as decerebrate or embryo forms) and revert to more robust, individual-level methods

## 3. Principles

What might, or should, the implied ethical features be in a policy-making context? There are many ethical concepts that might be relevant and could be applied as relevant principles for policymaking, including normativity, cognitivism, naturalism, prescriptivity, universalizability, justice, generic consistency, categorically imperative, reflective equilibrium, equipoise, and calculus. However, to narrow the field to what is achievable within a single paper, the following analysis shall consider the Seven Principles of Public Life (aka. Nolan Principles)-selflessness, integrity, objectivity, accountability, openness, honesty, and leadership-and their application to ascribing sentience.

### 3.1. Selflessness

The principle of selflessness asserts that ‘Holders of public office should act solely in terms of the public interest.’ While the Committee doubtless had in mind the personal interest of humans, it is worth considering how we might consider a wider concept of public interests in animal protection.

The principle of selflessness firstly highlights that the ascription of sentience, like other policies, is a normative matter in terms of its purpose and impact. The reason for ascribing sentience is to protect vulnerable animals who can experience suffering or other feelings, and to help society to avoid causing unjustified suffering or deprivation. This dictates an appropriate definition for sentience by relating it to experiences that matter. Sentience in this understanding is not *per se* a matter of perception or cognition (or language), but a matter of being able to have positive and negative experiences. For example, the SAWC defined animal sentience as: ‘the ability to have physical and emotional experiences, which matter to the animal, and which can be positive and negative’ [5] and the DEFRA-commissioned study defined sentience as ‘the capacity to have feelings’ [6].

On hedonic or teleological ethical approaches, these would include experiences that matter to the animal. We might also include experiences that relate to animals’ assessments of what matters. This would suggest we are concerned with valenced affective states (i.e., pain, pleasure etc.) or motivational states (i.e., preferences, frustration, anticipation etc.). In this, while we might see ascriptions as ultimately based on empathy, it is not necessary to be able to describe precisely “what it is like” subjectively to be a bat or how they experience “red”, but simply to recognise the evidence for valenced or motivational experiences.

Selflessness is, secondly, also about ensuring our ascriptions are not ones that benefit only ourselves, and in this context “ourselves” relates to our species. Policies should aim to protect all sentient animals. Specifically, selflessness requires policymakers to avoid unduly prioritising human interests in the exploitation of animals whose sentience is under question (notwithstanding those practices involving the harming of animals such as wildlife trade and intensive farming are drivers of threats to humans such as zoonotic pandemic emergence, climate change, pollution and biodiversity and ecosystem loss, which suggests that preventing suffering (in its own right) can also be expected to benefit humans).

Put simply, assessments on whether a species is sentient should not be affected by the purely financial implications of that assessment. Even if a practice such as live boiling is widespread and lucrative or enjoyable (or otherwise beneficial for humans), that should not affect whether the animals affected should be deemed sentient.

One potential solution to this is to recognize these partialities as post hoc exemptions, rather than trying to retrofit our ascriptions to suit them. In other words, policies can ultimatel sentience where the evidence supports it and then transparently factor economic and other concerns into the policies about how those animals are treated. While this still might not ultimately afford animals the protection needed, it at least locates debate in the right point in the policymaking process.

Indeed, we might go further and suggest that selflessness would prescribe giving animals the benefit of the doubt. There is an asymmetry in getting ascriptions wrong: it is likely, *ceteris paribus*, to be far worse to falsely deny sentience than to falsely ascribe it. So, without embracing an overly liberal ascription (e.g., to all lifeforms), policies can be expected to have a greater utility if there is a modest presumption of erring towards (rather than against) an assumption of sentience. It is selfless to give animals the benefit of the doubt, when doing so has relatively minor impacts on our own wellbeing (whether or not greater altruistic selflessness is to be expected of policymaking or policymakers themselves).

### 3.2. Integrity

A slightly different principle is that of integrity. Integrity in this context relates to recognising and avoiding and resolving any ‘inappropriate influence’ towards oneself, one’s relatives or other people or organisations in their work. We have considered biases towards humans’ non-moral interests under selflessness, which could manifest themselves as undue pressures from those who benefit from using animals in ways that would cause sentient animals suffering. There are also other potential influences that favour humans.

At its broadest, this principle suggests that we should not be unduly influenced towards ascribing sentience to ourselves above other animals. Applied to ascription, this means avoiding solipsism regarding oneself or human exceptionalism regarding one’s species and, more widely, avoiding systematic pro-human assumptions. It does not mean ignoring the plausible view that we can have greater confidence in (most) humans’ sentience than (most) other animals’, but it requires the greater confidence in human sentience should be the a posteriori result, rather than an inbuilt a priori assumption, of the methods.

Some quite subtle pro-human influences risk being structurally built into ascriptions insofar as ascriptions are based on extrapolations or analogical reasoning from ourselves, either in terms of associations or of explanations. Generalising from ourselves creates a risk that we are less likely or able to ascribe feelings we have personally never experienced or based on observable criteria that we lack. For example, I have not experienced being part of a shoal, vertical migration, pleustonic variation or having decentralized control of eight legs, and might therefore be less able to identify such behaviours as relevant to experiences.

Ascriptions similarly need to avoid predetermining conclusions by using differing criteria or evidential thresholds for humans (e.g., babies) versus other species, or relying on verbal reportage or other methods that cannot be applied to nonhuman species. Different ascriptions for different species can be justified if and only if there are relevant differences in the evidence relating to those animals. Ascriptions, therefore, need to extrapolate or analogise oneself in ways that avoid treating irrelevant (or “accidental”) differences between ourselves and other animals as relevant, and ignore as irrelevant any characteristics that we do not see as having a non-chance association with and/or explanation for our experiences, such as eye colour, language, and genetic species membership *per se*.

### 3.3. Objectivity

Objectivity is explained as ‘act(ing) and tak(ing) decisions impartially, fairly and on merit, using the best evidence and without discrimination or bias.’ There are two complementary elements to this: recognising the best evidence available and avoiding partiality, unfairness, discrimination and bias. Basing ascriptions on the best evidence is both a positive matter of using the most relevant, informative and reliable data available, and a negative matter of avoiding biases that are based on irrelevant evidence. The use of the best evidence may help make decisions “on merit”, so long as it does not bring in (and hide) other biases.

Making ascriptions fairly includes minimising undue species biases. In addition to the pro-human biases considered as matters of Selflessness and Integrity, other biases include partiality for other animal species based on metaphysical concepts, common affections for animals such as dogs and cats, and prejudices against animals such as spiders and flies [31]. other biases can include cognitive biases, including confirmation bias, cognitive inertia and the avoidance of cognitive dissonance or guilt by engineering ascriptions to justify our previous or ongoing (mis)treatment of animals. Biases may also be affected by the individual making the assessment e.g., [32,33].

Such objectivity requires us to employ the same methods of ascription to evaluate sentience in other species as we would want others to evaluate sentience in oneself in situations. We might imagine hypothetical heuristic scenarios, such as an angel or alien arriving on Earth and having to ascertain our sentience or us having to regulate under a Rawlsian/Rowlandsian veil of ignorance, where the imagined veil precludes knowledge of which species is our own. Such an approach would not logically use species as a criterion in its own right, since to be consistent, that criterion would be not “human” but “the same species as me”, which is a criterion that would be biased against us when applied by other species and nonsensical to apply behind a veil of ignorance as to one’s species.

More positively, best evidence is an evaluative concept. The use of best evidence requires the evaluation of the data that support and weaken the ascription of sentience. The warrant for an ascription is a matter of the evidence’s weight (e.g., number of studies), strength (e.g., similarity), reliability (e.g., of an association), and relevance (e.g., as an explanation) so it is more than simply quantifiable observations. It is further a matter of “triangulation” across all evidence, basing ascriptions on the cumulative and interactive effect of multiple data, insofar as our ascriptions form a “web” of interacting explanations, analogies and ascriptions. In this way, all ascriptions affect each other, insofar as the totality should aim to be cogent, internally consistent and mutually supportive. The “denser” the web of evidence, the stronger the grounds for analogy.

These are matters of degree, which suggests that warrant and confidence should also be continuous variables. Degrees of confidence would range in proportion to the evidence from near-absolute (in oneself) to negligible (in, we might expect, inanimate abiotic objects). We might relate confidence to the strength and weight of evidence, and to the degree of relevant similarities. Recognising that there are degrees of confidence could also allow the justification of affording a lesser degree of protection to animals in whose sentience we are less certain (e.g., using fruit-flies instead of zebrafish in scientific research), while still protecting species with weaker evidence from harms for trivial purposes such as live boiling of crustaceans in haut cuisine cooking or placing insects in simulated predator-prey situations for entertainment.

Stronger, more reliable evidence (e.g., meta-analyses of scientific studies) might be afforded more weight than weaker evidence (e.g., personal anecdotal experiences). The principle of using the “best evidence” therefore promotes the use of scientific evidence, and the need for the best evidence creates a responsibility to invest in research to obtain, find, collect and evaluate data. This includes funding studies looking at different species and reviewing that data. It also includes research to develop better methods of ascription, the result of which constitute ever better evidence.

However, the concern for using the best evidence does not mean that policymakers need perfect evidence or should limit the evidence to scientific data. It means policies should be based on the best evidence available. In some case, this may be non-scientific evidence which, if ignored, would leave animals unprotected. In the absence of robust scientific data, the best evidence may well be, in such cases, the perceptive responses of unconflicted and empathetic people. Conversely, the use of other sources of evidence and approaches does not reduce the value of better evidence; initial ascriptions should be seen as an interim protective policy until better evidence is obtained.

The use of scientific evidence is also not a solution to all partiality or biases. Firstly, the use of evidence may be subject to biases: as described above, evidential requirements and interpretations are normative matters. Secondly, the scientific approach itself has built-in ethical assumptions and implications that may build in structural biases, which may be unnoticed or unremarked if they are widely held biases [34,35].

For example, ascriptions may draw on studies that observe associations between contexts and behaviours such as the placement of an acidic chemical on an antenna. Such studies often use frequentist statistical methods to distinguish chance from non-chance associations. In the wider scientific approach, this is a logical safeguard to reduce the chance of erroneous positive findings. However, such statistical methods can create asymmetrical biases: a *p*-value of 0.95 could be taken to represent a 5% likelihood of a Type I error that wrongly identifies a relationship that supports sentience, but a 20% chance of a Type II error that would wrongly miss an association that would supports it [36]. This asymmetry results in a bias against finding evidence of sentience, from which policymakers might fallaciously infer as insentience. Presuming null hypotheses, requiring statistically-significant data, or discounting “simpler” explanations that do not require ascribing sentience (e.g., nociceptive reflexes), effectively favour the non-ascription of sentience. As such, in policy terms, they favour whatever is the default non-scientific presumption on how animals are treated. Another well-known bias is Morgan’s canon, which prescribes assuming behaviours are mediated by “lower” cognitive processes, which may be taken to imply processes not supervening upon experiences.

These structural biases of current scientific methods are the opposite of the bias towards ascription that might be expected to minimise suffering. In this case, the scientific approach runs counter to at least one moral approach that the Nolan Principles might be seen as prescribing. As such, we might consider that scientific data should be used in ways that counter the inbuilt bias (and at least do not add to it), for example, by more “liberal” generalisations and lower thresholds of what counts as “sufficient” evidence.

Overall, there is a risk of using scientifically-justified methods to make morally-unjustified decisions. This brings a risk that policymakers believe or claim they are being objective and impartial by using science, but actually have built in biases in the decisions of how to use science. If we ignore these biases, we might blindly believe policies are being objectively scientific, while inadvertently favouring particular ethical views. 

### 3.4. Accountability

Policymakers “are accountable to the public for their decisions and actions and must submit themselves to the scrutiny necessary to ensure this.” This has two elements. The first is accountability in that decisions must be made. The second is that they are responsible for their decisions, including decisions to make decisions in a particular way (who, what, when, why, how much and how). Policymakers are specifically accountable for a decision to rely on science or to “outsource” decisions on what animals are sentient to scientists: such decisions do not absolve policymakers of their responsibility, and policymakers need to discharge that responsibility by being aware of the implications of how they use scientific data and experts. The third is that those decisions should be considered subject to correction.

Policymakers are responsible for their decisions, including their decisions not to make, or to defer making, a decision. A decision not to make an ascription is a decision. Policymakers are then accountable for any resultant suffering if the animals are, in point of fact, sentient. For example, the exclusion of decapods in UK animal welfare legislation has permitted the suffering of lobsters through inhumane killing methods during that time.

Some policymaking might explicitly reject the consideration of certain animals’ sentience outright (most often for policies within an overarching policy framework that prespecifies a particular scope, e.g., ignoring cephalopod sentience in secondary legislation under the Animal Welfare Act 2006). Others might be due to industry pressure, and policymakers are also, of course, accountable for decisions to base policies on non-scientific factors such as stakeholder pressure to delay or avoid protecting certain animals.

However, decisions might be more subtly avoided or deferred by policies that require scientific evidence or scientific consensus. This excludes animals that have simply not been studied yet. It also effectively brings the structural scientific biases into the policymaking. In particular, scientific approaches legitimately tolerate uncertainty, for example by presuming null hypotheses until presented with evidence that is sufficient under a statistical definition, usually in relation to confidence intervals.

In comparison, policymakers cannot reserve judgement without real-world implications. Policymakers need to make decisions about how to treat animals, such as cephalopods and decapods, under conditions of scientific uncertainty. An “appeal to ignorance” might sometimes be politically expedient, but it is not, in this case, a legitimate excuse for allowing unnecessary suffering. This holds even in the absence of studies, insofar as it creates a responsibility to either change the default position (e.g., to a more precautional approach) or the evidential requirements (e.g., to allow weaker evidence) - *as well as* any responsibility to ensure the evidence gaps are filled. This reinforces the recognition that policymakers have a moral imperative to assess animal sentience on the evidence available.

When policymakers decide to base the policy on science, they are further accountable for their decisions on how that science is used within the policymaking framework. Firstly, policymakers need to ensure that scientific methodologies are used for the evidential questions to which they are suited–associations between observations–but that policies also use other concepts of justification for our decisions, such as ethical justification. Secondly, policymakers are responsible for where they set evidential requirements. In particular, if policymakers always require a particularly high level of scientific evidence before ascribing sentience (and require an ascription of sentience before protecting animals), then they effectively decide not to protect animals until that evidence is available (if it ever can be) and therefore effectively decide to continue a default or status quo policy approach that allows humans to cause suffering.

One implication of policymakers’ accountability for the implications of their decisions is that, on a consequentialist approach, policymakers have a duty to use a method of ascription which is expected to minimise overall suffering. Such an approach needs to minimise the risks of under-ascribing (when not outweighed by significant other concerns for which they are responsible). Such a policy would at least be one that ascribes evidence on the basis of a more modest level of “sufficient” evidence rather than proof, and arguably would be one that ascribes on a default assumption of animal sentience.

One additional responsibility for accountable policymakers is to accept decisions might be wrong and need refinement or correction. This may feel challenging for those under lay media scrutiny. Nonetheless, this is an important aspect of ascribing sentience. We should see ascriptions as plausible views that need to be reviewed and our confidence amended subject to subsequent new evidence. These revisions should not “change our belief” in a binary way (which is a high demand on any one piece of information) but might proportionately modulate our confidence in an ascription in a proportionate way, thereby avoiding the risk of it being too easy to throw an ascription back into complete question. This is more a matter of corrigibility than defeasibility.

The refinement of our ascriptions should be an iterative process as new data is obtained and considered. In Bayesian terms, this would consider (or conditionalize) new data in light of the prior confidence in a given ascription, to generate a revised confidence in the ascription. The initial prior confidence might legitimately be based on non-scientific assessments of explanations for behaviour, see [37,38], and then be refined as additional scientific evidence is taken into account. This would be iterative by building on the previous confidence plus the new evidence. Each new iteration should also refine the confidence in the methods and criteria on which they are based as part of the overall web of evidence and ascriptions.

### 3.5. Openness

To ascribe in an open and transparent manner, policymakers should be open and transparent about which data are used, when and how science is used, the ethical bases of the method of ascription, the implications of that approach, and of course the conclusions reached. The DEFRA-commissioned study was released, albeit after some active lobbying by non-governmental animal welfare organisations such as Crustacean Compassion. Articulating the underlying ethical and methodological aspects of the approach to ascription might help to provide a framework for discussions. This is particularly valuable to ensure ethical views do not bias policies in hidden and non-systematic ways.

Articulating the underlying methods and ethics is also useful to improve the debate. Hidden ethical views appear to provoke debates between focus and lobby groups who have access to the same scientific data but reach different conclusions (which sometimes leads them to cite different subsets of those data). Without understanding how data are used, these debates are unproductive and potentially intractable. Discussing the ethical grounds on which analogical or abductive inferences are justified is a way to foster discussion, as ideas people can understand metaphorically or empathetically. This also entails guarding against assuming animals’ behaviour is explained by the “lowest” psychological mechanisms, as citizens may well find experience-based explanations of animals’ behaviour, that relate to their own behaviour and experiences, more understandable and accessible than convoluted explanations that try to avoid ascribing experiences.

### 3.6. Honesty

Our policy on ascriptions should obviously avoid conclusions that are believed to be likely to be untrue. Our ascriptions should be based on beliefs that are as well founded and as confident as possible. This means evidential requirements need to be feasible (and this is also an ethical principle, as the converse of “ought implies can”), operationalizable within the limitations of current evidence, and applicable to reality as it appears to us from all the evidence available. This means not requiring a standard of proof or degree of certainty that would necessitate a perfect ability to imagine what other animals’ experiences feel like, or data that do not exist yet.

The principle of honesty does not mean that policymakers may only make statements about which they are absolutely and incontrovertibly certain. Inevitably policymakers must make statements based on the best information available and a legitimate interpretation. We must further accept that some will be justifiable errors. The principle of honesty can be understood as prescribing that policy positions and conclusions should be based on beliefs that are justifiable, with that justification being both evidential and ethical.

### 3.7. Leadership

The principle of leadership “piggybacks” on the other principles, in the sense of promoting them. Nonetheless, we might consider additional leadership qualities such as being inclusive (i.e., avoiding exclusionary biases), respectful and transformational. Policymaking in this context needs to overcome conservative inertia and biases, and avoid placing undue barriers against recognising the sentience of other species. 

More widely, the leadership on this issue should look to transform our relationship with sentient animals. As the United Nations Secretary General stated in the UN’s Harmony with Nature report, “A first step to recognizing the rights of Nature is the recognition that non-human animals are sentient beings, not mere property, and must be afforded respect and legal recognition” [39].

## 4. Conclusions

Policymakers face an unusual challenge in determining how to ascribe sentience to nonhuman animals, and this challenge cannot be avoided or overcome by the simple naïve use of scientific methods. Scientific data are important for determining observable associations and how widely they can be generalised across taxa. However, they cannot determine how those data are used, what is done in the absence of sufficient data or how to overcome the biases that are built into the scientific methods. The ascriptionof sentience to nonhuman animals is a challenge loaded with ethical dimensions that need transparent articulation, careful examination, and open discussion. The consideration of basic ethical principles for policymaking may help to avoid the more extreme errors and, one hopes to minimise suffering for sentient animals.

## Data Availability

The study did not report any data.
